# Detecting Chromosome Instability in Cancer: Approaches to Resolve Cell-to-Cell Heterogeneity

**DOI:** 10.3390/cancers11020226

**Published:** 2019-02-15

**Authors:** Chloe C. Lepage, Claire R. Morden, Michaela C. L. Palmer, Mark W. Nachtigal, Kirk J. McManus

**Affiliations:** 1 Department of Biochemistry & Medical Genetics, University of Manitoba, Winnipeg, MB R3E 0J9, Canada; lepagec@myumanitoba.ca (C.C.L.); mordenc1@myumanitoba.ca (C.R.M.); palmerm3@umanitoba.ca (M.C.L.P.); Mark.Nachtigal@umanitoba.ca (M.W.N.); 2 Research Institute in Oncology & Hematology, Winnipeg, MB R3E 0V9, Canada; 3 Department of Obstetrics, Gynecology, and Reproductive Sciences, University of Manitoba, Winnipeg, MB R3E 3P5, Canada

**Keywords:** chromosome instability, cancer, intratumoral heterogeneity, single cell approaches, quantitative imaging microscopy

## Abstract

Chromosome instability (CIN) is defined as an increased rate of chromosome gains and losses that manifests as cell-to-cell karyotypic heterogeneity and drives cancer initiation and evolution. Current research efforts are aimed at identifying the etiological origins of CIN, establishing its roles in cancer pathogenesis, understanding its implications for patient prognosis, and developing novel therapeutics that are capable of exploiting CIN. Thus, the ability to accurately identify and evaluate CIN is critical within both research and clinical settings. Here, we provide an overview of quantitative single cell approaches that evaluate and resolve cell-to-cell heterogeneity and CIN, and discuss considerations when selecting the most appropriate approach to suit both research and clinical contexts.

## 1. Introduction

Genome instability is an enabling feature of cancer [1], and it refers to a state of increased mutations, copy number changes, and epigenetic alterations within a cell [2]. Genome instability exhibits critical roles in cancer initiation, progression, evolution, and drug resistance (reviewed in [1,2,3,4,5]), and is characteristic of virtually all cancer types [6,7,8]. Traditionally, genome instability has been categorized into three distinct forms: (1) microsatellite instability (MSI); (2) CpG island methylator phenotype (CIMP); and (3) chromosome instability (CIN) [2]. While MSI arises due to defects in DNA mismatch repair genes (*MLH1*, *MSH2*, *MSH6*, and *PMS2*) that underlie DNA mismatches, as well as expansions and/or contractions of repetitive DNA sequences termed microsatellites [2,4] (reviewed in [9]), CIMP is characterized by extensive DNA methylation (CpG dinucleotides) within promoter regions, leading to transcriptional silencing [2] (reviewed in [10]). CIN is a third form of genome instability, and is defined as an increase in the rate at which whole chromosomes or large parts thereof are gained or lost [2,11,12]. Collectively, all three forms of genome instability contribute to cancer pathogenesis by altering the expression and/or encoded functions of key genes, and thus they are significant contributors to the aberrant genetic and epigenetic landscapes contained within cancer cells [1,13]. CIN has a particularly profound impact on genome stability by inducing simultaneous and ongoing copy number changes in large cohorts of genes (e.g., oncogenes, tumor suppressor genes, DNA repair genes and apoptotic genes) that promote oncogenesis [14,15,16,17]. Thus, CIN is a dynamic phenotype that increases the probability of acquiring the myriad of genetic changes that are required to initiate and drive the development and progression of cancer [18,19,20]. Due to the pervasive nature of CIN within cancer, this review is focused on detailing CIN and the techniques that are used to detect and evaluate CIN in both research and clinical settings.

**Figure 1:**
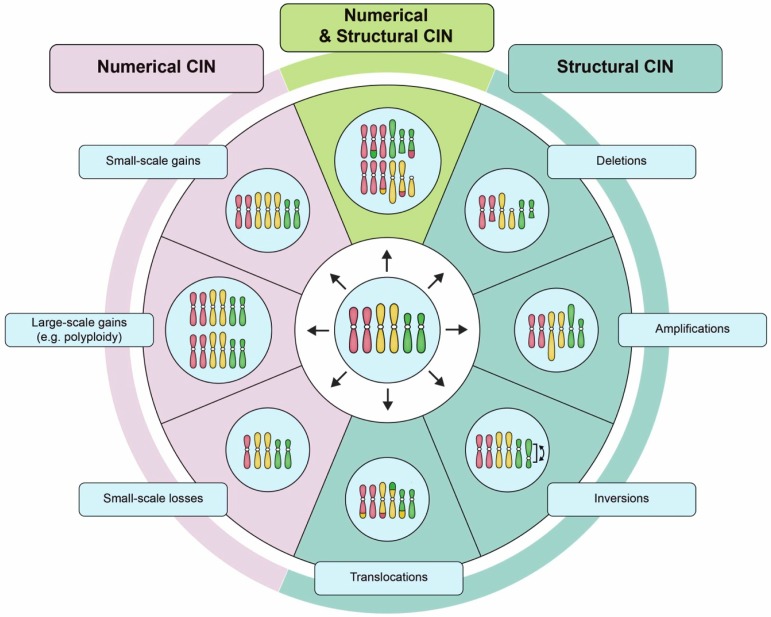
Examples of numerical chromosome instability (CIN) and structural CIN. A schematic depicting examples of the types of karyotypic changes associated with either numerical CIN (N-CIN) or structural CIN (S-CIN). Note that to accurately define CIN within a given population requires multiple distinct karyotypes to be identified as a single aberrant karyotype only defines a state, and not a rate. For illustrative purposes, the starting diploid cell (center) only contains three pairs of chromosomes (i.e., a partial karyotype). N-CIN involves whole chromosome gains or losses, including both small-scale changes that result in aneuploidy, as well as large-scale polyploidization events. S-CIN includes partial chromosome deletions, amplifications, inversions, or translocations (ranging in size from single genes to entire chromosome arms). These different classes of N-CIN or S-CIN are often combined to produce complex karyotypes that evolve over time. However, techniques for evaluating CIN typically only detect a subset of these karyotypic changes.

**Figure 2:**
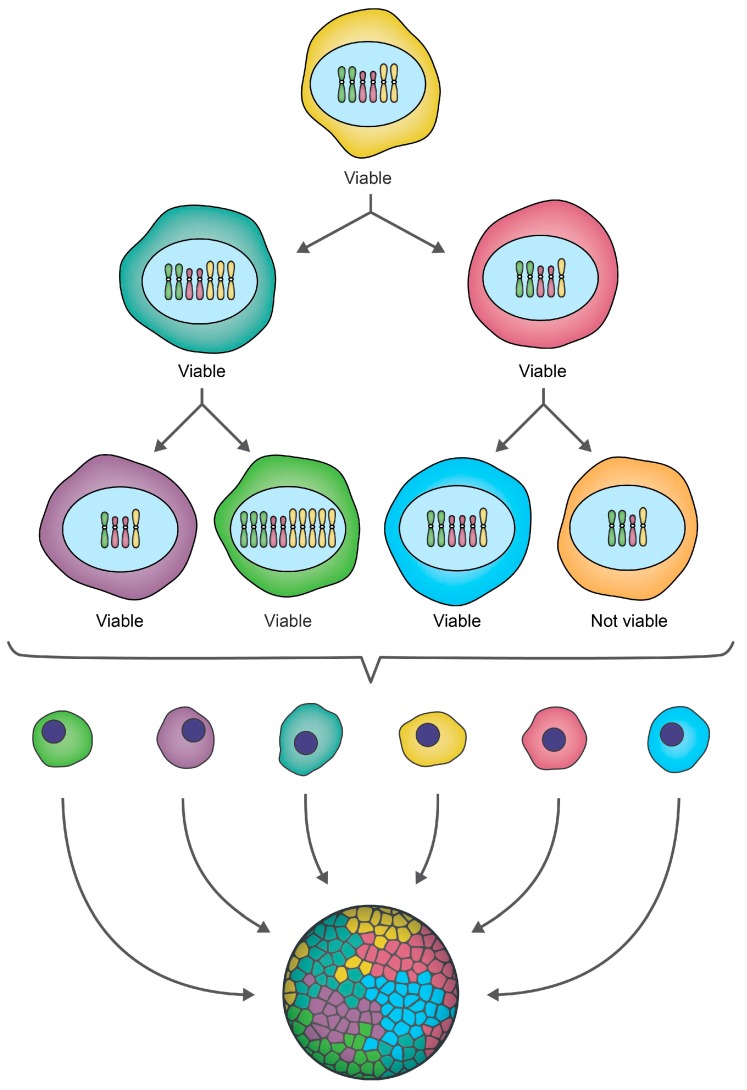
CIN drives ongoing karyotypic heterogeneity within cellular populations. A schematic depicting a hypothetical example of CIN within an initial cell that for illustrative purposes contains only three pairs of chromosomes (i.e., a partial karyotype). As this cell undergoes two rounds of cellular division, chromosomes are gained or lost, producing a heterogeneous population of genetically distinct daughter cells, which is referred to as intratumoral heterogeneity in a cancer context. Some karyotypic changes may not be compatible with cell viability, as indicated by the orange cell which is lost from the population. Note that while this example focuses on small-scale gains/losses of whole chromosomes (N-CIN), chromosome complements may also evolve via increases in ploidy (N-CIN) or structural chromosome changes (S-CIN) (see Figure 1), and often include a combination of both N- and S-CIN.

The chromosomal alterations associated with CIN can be broadly classified as either: (1) numerical (N-CIN), involving gains and/or losses of whole chromosomes [12]; or, (2) structural (S-CIN), involving amplifications, deletions, inversions, and translocations of chromosomal regions that can range in size from single genes to whole chromosome arms [2,12,21] (Figure 1). Conceptually, the ongoing chromosome gains, losses, or structural alterations associated with CIN promote the production of genetically distinct (i.e., heterogeneous) populations of daughter cells (Figure 2). Thus, within the context of cancer, CIN increases intratumoral heterogeneity that under certain conditions may confer a selective growth advantage (e.g., increased cell proliferation, metastatic potential, or intrinsic drug resistance) to a subpopulation of cells [22]. Under certain selective pressures, like chemotherapy, cells harboring specific growth advantages, such as drug resistance, will continue to proliferate and may ultimately produce a highly aggressive or drug-resistant tumor [23]. Thus, it is not surprising that CIN is frequently associated with disease recurrence and poor patient outcomes [14,22,24,25,26,27,28,29,30,31,32]. Paradoxically however, CIN can also be associated with more favorable outcomes, but typically only in certain cancer contexts [33,34]. These contradictory findings may be rationalized by differences in the rates of chromosomal changes observed in different cancer types, cancer subtypes and even within individual tumors [2,35]. It has been suggested that extreme levels of chromosomal changes, or high rates of CIN may not be compatible with viability and thus, cancer cells with inherently high levels of CIN die and are lost from the tumor cell population [36,37]. In this regard, CIN may represent a therapeutic vulnerability that can be leveraged to induce cancer-specific cell killing [38,39,40,41]. Thus, identifying CIN and the causative genes and pathways are of tremendous clinical interest, as they may represent valuable diagnostic/prognostic biomarkers with implications for healthcare delivery and treatment decisions.

It is generally accepted that the aberrant genes underlying CIN (i.e., CIN genes) normally function within key pathways that orchestrate chromosome dynamics. Indeed, many human CIN genes have been identified to encode functions in DNA replication and repair [12,42], centrosome duplication [43,44], mitotic spindle dynamics [44], kinetochore-microtubule attachment [45,46], sister chromatid cohesion [47,48,49,50], chromosome segregation [51], and cell cycle checkpoints [52,53,54,55]. Since many of these pathways are evolutionarily conserved [56], cross-species approaches have been successfully employed to identify human CIN genes, and they further predict that many additional CIN genes have yet to be discovered in humans. For example, Stirling and colleagues performed a comprehensive assessment of all budding yeast genes, and determined that ~11.5% are CIN genes (692/~6000 genes) [56], which when extrapolated to the human genome, equates to ~2300 genes. Interestingly, while many of the yeast genes encode functions within the pathways listed above, many additional CIN genes encode functions within less intuitive pathways that are not directly related to chromosome dynamics, such as proteasome function, transfer RNA (tRNA) synthesis, or lipid synthesis. Collectively, these observations suggest that many CIN genes exist within humans that have yet to be identified and functionally characterized in the contexts of CIN and cancer pathogenesis. Consequently, ongoing research efforts are currently focused on identifying novel CIN genes [51,57], evaluating their impacts in patient outcomes [30] and treatment responses [58], and determining their potential as novel therapeutic targets [38,59]. Thus, the ability to accurately identify and evaluate CIN and CIN genes is critical within research and clinical contexts.

## 2. The Importance of Single-Cell Approaches for Evaluating CIN

Many traditional techniques, like comparative genomic hybridization (CGH), single nucleotide polymorphism arrays, polymerase chain reaction-based methods, or flow cytometry, employ population-based averaging to identify gene copy number changes or aneuploidy within pooled samples, and erroneously equate these findings with CIN [60,61,62,63,64]. However, CIN refers to an ongoing rate of change that drives cell-to-cell heterogeneity (Figure 2) rather than a static state such as aneuploidy. Unfortunately, the population averaging associated with many of these classical studies effectively masks the cell-to-cell heterogeneity contained within the pooled samples, rendering it impossible to accurately assess CIN [65]. This concept is more clearly demonstrated in a study by Bakker et al., where CIN was analyzed in mouse T-cell acute lymphoblastic lymphomas using both CGH (population-based approach) and single cell whole genome sequencing (sc-WGS; detailed in Section 6.3) [65]. Comparison of these approaches revealed that CGH, a common technique used to identify aneuploidy and/or copy number alterations, failed to detect the level of karyotypic heterogeneity and CIN identified by sc-WGS [65]. These findings underscore the importance of assessing CIN at the single cell level using techniques that are capable of capturing the full spectrum of heterogeneity and CIN existing within a given sample.

In general, there are two central approaches that are used to assess CIN: (1) tracking chromosome numbers within a single cell and its progeny over time, and (2) quantitatively assessing cell-to-cell heterogeneity within a given population (Table 1) [2]. A fundamental benefit of the first concept is that it allows for the calculation of an exact rate of chromosomal gains/losses over time, but this requires the use of experimental approaches, like live cell approaches and transgenic chromosome markers (Section 3) that do not adversely impinge upon cell proliferation or viability. The second concept operates under the premise that CIN drives karyotypic heterogeneity, which in a given cellular population, can be readily detected through the use of quantitative, single cell approaches [2], including cytogenetic approaches (Section 4), quantitative and high-throughput imaging cytometry (Section 5), and single cell genomics (Section 6). Due to the inherent complexities of the first approach, the latter approaches are more commonly employed as they are easily adapted to traditional endpoint analyses (live or fixed), and they can be readily employed on a wide variety of samples including precious clinical specimens. Below, we describe each of these approaches, discuss their fundamental benefits and limitations, and present recent representative examples of how they have been applied within research and/or clinical settings.

## 3. Live Cell Approaches and Transgenic Chromosome Markers

To assess CIN by determining a rate of chromosomal changes mandates the use of techniques that are capable of analyzing continuously growing cultures initiated from a single cell, at regular time intervals (i.e., serial sample analyses). Alternatively, single-cell tracking can be employed to simultaneously monitor multiple cells and their progeny within a given experimental population. In either case, determining the rates of chromosomal changes (CIN) necessitates the use of techniques that do not adversely impact cell viability or proliferation, and thus live cell imaging is most often employed. Visualization of chromosomes can be achieved in several ways, such as fluorescent labeling of chromatin-associated proteins, fluorescent operator/reporter systems, fluorescently labeled artificial chromosomes, and modified gene editing systems, all of which are detailed below.

### 3.1. Fluorescent Labeling of Chromatin-Associated Proteins

Chromosome labeling for live cell imaging can be achieved by using a genetically-encoded fluorescent tag to detect histones or other chromatin-associated proteins (Table 1) [66]. As this approach uniformly labels all chromosomes, it will not allow one to track gains or losses in a specific chromosome, and thus, does not measure CIN per se. Rather, this approach enables the assessment of chromosome dynamics as cells progress through mitosis that may reveal aberrant mitotic events including chromosome congression (to the metaphase plate) or segregation (from the metaphase plate) errors, anaphase bridges, chromosome breakages and chromosome decompaction, which are all phenotypes associated with CIN. For example, Kanda and colleagues employed green fluorescent protein (GFP)-tagged histone H2B to observe the dynamics and segregation of chromosomes and double minutes within human HeLa cells, and noted the utility of this technique in detecting lagging chromosomes, chromosome fragmentation, and aberrant chromosome condensation states (based on GFP signal intensity) [66]. More recently, Thompson et al. evaluated the frequencies of mitotic defects (lagging chromosomes and anaphase bridges) within karyotypically unstable (CIN-positive) human cell lines in comparison to karyotypically stable (CIN-negative) aneuploid cell lines, in order to investigate the relationship between aneuploidy and CIN [67]. As indicated above, however, the reliable enumeration of chromosomes is not feasible using this technique, and thus they subsequently employed fluorescence in situ hybridization (FISH) to quantify N-CIN within these samples. Finally, although this approach has traditionally relied on the manual inspection of images, which is laborious and time-consuming, software-driven automation and analytics are now possible using single cell tracking tools inherent within many image capture software packages [68].

### 3.2. Fluorescent Reporter/Operator Systems

Fluorescent reporter/operator systems enable the quantification of N-CIN within live cells, and they traditionally consist of a fluorescently-tagged DNA binding protein (“reporter”) that binds to a DNA element (“operator”) stably integrated within the genome at a defined chromosomal locus. For example, the Lac operator (*LacO*) system employs a fluorescently tagged (e.g., GFP) Lac repressor protein (LacI) to bind to a *LacO* array, and it presents as a single fluorescent focus that is easily detected using standard fluorescence microscopy [69,70,71]. The number of fluorescent foci present within a cell can be used as a surrogate marker for chromosome copy numbers, and this can be monitored through multiple rounds of cell division. Thus, both population heterogeneity and the temporal dynamics of copy number gains or losses can be quantified to evaluate N-CIN. The utility of this assay was demonstrated by Thompson et al., who employed red fluorescent protein (DsRED)-LacI labeling of chromosome 11 as part of a multiplexed high-content approach for evaluating CIN within a research context [70]. However, this approach is incapable of assessing S-CIN and is only informative for the chromosome harboring the *LacO* array, thus, events involving non-labelled chromosomes are not detected. In addition, this approach assumes that introducing an array of foreign DNA into the host genome does not itself impact chromosome stability (e.g., by disrupting critical genes or by generating a fragile site that is prone to breakage/structural alterations [72]). Finally, this approach involves the generation of a transgenic cell line, which requires cells to be able to accept and tolerate the introduction of the *LacO* array, and that they remain stable over prolonged periods of time, such as karyotypically stable transformed or immortalized cell lines. Nevertheless, and once generated, these cell models are ideally suited to high-throughput screens, and they can be multiplexed with quantitative imaging microscopy (QuantIM) assays (see Section 5.1).

### 3.3. Human and Mouse Artificial Chromosomes

Rather than introducing a transgenic marker into an endogenous chromosomal locus, a related approach involves the use of human or mouse artificial chromosomes (HACs or MACs) engineered to contain an informative reporter gene (e.g., GFP) to enable the assessment of HAC/MAC copy number changes via flow cytometry or QuantIM (Table 1) [73]. HACs/MACs include centromeric sequences that form functional kinetochores, and they rely on the same segregation machinery as endogenous chromosomes [74], and thus an increased rate of HAC/MAC copy number changes is indicative of an increased rate of whole chromosome missegregation, or N-CIN. While these systems would theoretically allow for the assessment of either gains or losses of a HAC/MAC, to date, they have primarily been designed to assess chromosome losses [75,76]. For example, Lee et al. employed HACs conferring GFP expression coupled with flow cytometry to evaluate the rate of HAC loss (i.e., CIN) in response to various chemotherapeutic agent treatments [77]. A fundamental limitation of HACs/MACs is that they do not directly detect changes involving endogenous chromosomes, and consequently they are unable to distinguish the rate at which specific chromosomes are gained or lost. Instead, these approaches assume a consistent rate of missegregation for all endogenous chromosomes that is equivalent to the rate of HAC/MAC missegregation. Interestingly, MACs are more stably maintained than HACs in some cell types, suggesting HACs (and even MACs) may have an inherent level of instability in certain contexts [78]. Additionally, as with other approaches that require introduction of foreign genetic material, HAC/MAC-based systems are only suitable for research-based applications and are likely to be most effective as preliminary screening tools.

### 3.4. Modified Gene Editing Systems

To date, few traditional approaches are capable of resolving S-CIN within live cells; however, emerging approaches are being employed to visualize specific loci employ gene editing technologies, including zinc finger nucleases (ZFNs) [79], transcription activator-like effector nucleases (TALENs) [80], and CRISPR/Cas9 systems (Table 1) [81]. In general, and for standard gene editing purposes, these methods are comprised of an endonuclease that is directed to a specific locus via a target recognition sequence. In ZFN and TALEN systems, the endonuclease activity and target recognition are provided by a single protein [82,83], while CRISPR typically employs the Cas9 endonuclease and RNAs (often a single guide RNA) for gene targeting [84]. To visually assess CIN, all three approaches have been adapted by replacing the endonuclease activity of the ZFN, TALEN, or Cas9 protein with a fluorescent tag (e.g., green or red fluorescent proteins) [79,80,81]. Thus, a specific genomic locus can be ‘probed’ in a manner similar to FISH, but with the added benefit of being able to assess copy number changes of a specific locus in live cells. The CRISPR-based approach offers enhanced versatility in the types of sequences that can be assessed by varying the number and types of guide RNAs employed. As an emerging technology, CRISPR/Cas9 systems have not yet been employed to assess CIN, but with further advancements, may prove a promising approach with unique benefits. For example, in human cell lines CRISPR/Cas9 has been employed to label repetitive DNA, non-repetitive DNA and whole chromosomes [81,85,86], which would theoretically enable the detection of both N-CIN and certain types of S-CIN, such as translocations or large insertions/deletions. However, like the transgenic chromosome markers, these approaches will require extensive development and validation prior to experimental execution, and thus they are ideally tailored to research-based contexts.

## 4. Cytogenetic Approaches

**Figure 3:**
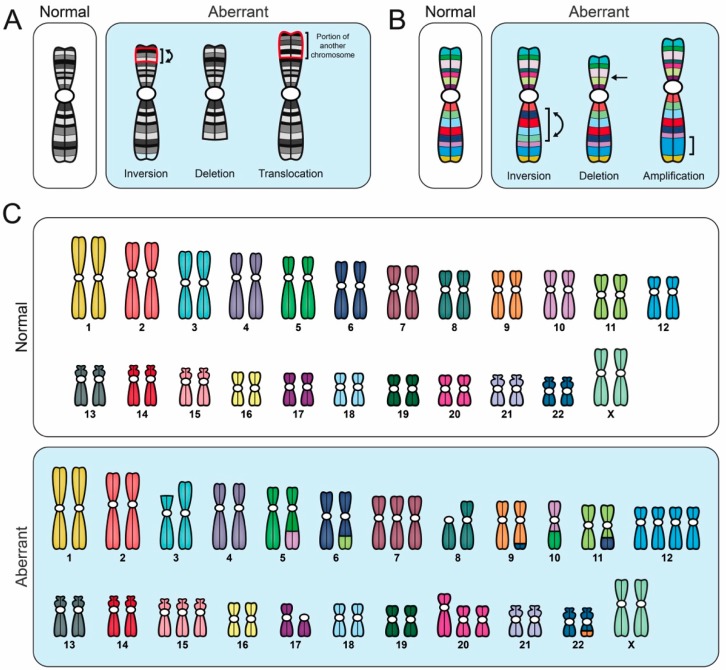
Comparison of different cytogenetic techniques used to evaluate CIN. Schematic illustrating various aberrant karyotypic events detected using specific cytogenetic techniques, all of which are performed on mitotic chromosome spreads. Importantly, to accurately identify CIN (i.e., cell-to-cell heterogeneity), numerous distinct aberrant events must be identified within a given population; a single aberrant karyotype that is stable within the population does not constitute CIN. (**A**) G-banding and inverted DAPI counterstaining enable full karyotypic assessment, and they identify individual chromosomes based on their unique banding pattern. Aberrant banding patterns are suggestive of structural abnormalities (i.e., S-CIN). (**B**) Multiplex-banding (M-banding) fluorescence in situ hybridization (FISH) employs multicolored probes generated for a specific chromosome or region, and they can be used to identify aberrant intrachromosomal events like inversions, deletions or amplifications (i.e., S-CIN). (**C**) Spectral karyotyping (SKY) employs multicolored probes to ‘paint’ each chromosome a unique color, enabling a full karyotypic assessment. SKY can detect chromosome copy number changes (gains or losses), interchromosomal translocations and some intrachromosomal events (large deletions and amplifications), but does not readily detect intrachromosomal inversions.

Cytogenetic approaches are widely employed within research and clinical settings, and are often capable of detecting N-CIN and S-CIN (Figure 3). Typical approaches include karyotypic analyses (Section 4.1), FISH (Section 4.2), spectral karyotyping (SKY) and multiplex-banding (M-banding) techniques (Section 4.3), each of which is detailed below.

### 4.1. Karyotypic Analyses

Conventional karyotyping involves analyzing Giemsa banding (G-banding) or inverted DAPI (4′,6-diamidino-2-phenylindole) staining patterns along metaphase chromosomes (Table 1) [87]. To perform standard karyotyping, mitotic chromosome spreads are generated that are subsequently counterstained (Giemsa or DAPI) to produce highly reproducible and chromosome-specific banding patterns. Typically, 25–50 mitotic chromosome spreads are assessed and the most frequently observed (modal) karyotype is determined and presented. Unfortunately, this single representation is incapable of describing any population heterogeneity that may exist, and thus they cannot be used to assess CIN. However, if all karyotypes from a given population are included in the analyses, G-banding and inverted DAPI staining are highly efficient at uncovering population heterogeneity, and they are ideally suited to identify N-CIN and S-CIN, provided the aberrant events (e.g., amplifications, insertions, deletions, inversions, translocations) are large enough and involve multiple bands (typically ≥1 megabase (Mb) in size) (Figure 3) [87]. Thus, an inherent limitation in assessing S-CIN is that events <1 Mb in size may be missed. Further, the ability to identify aberrant chromosome numbers or banding patterns mandates the cells be actively progressing through the cell cycle, or be pharmacologically induced to divide, so that mitotic chromosome spreads can be generated. Consequently, karyotypic analyses are incapable of assessing CIN in cells undergoing endoreduplication (i.e., repeated rounds of replication in the absence of mitosis) [88], and are not readily applicable to clinical samples like formalin-fixed paraffin-embedded tissues [87]. Despite these limitations, karyotypic analyses are amenable to many research and clinical settings (e.g., cell lines and patient samples), and within various cancer contexts, particularly hematological cancers [47,89]. For example, Chin and colleagues recently employed G-banding to identify chromosome aberrations (N- and S-CIN) in cells that are isolated from the peripheral blood of children with acute lymphoblastic leukemia [89], while Babu et al. employed inverted DAPI staining to assess N-CIN within Hodgkin lymphoma cell lines [47].

### 4.2. Fluorescence In Situ Hybridization

**Figure 4:**
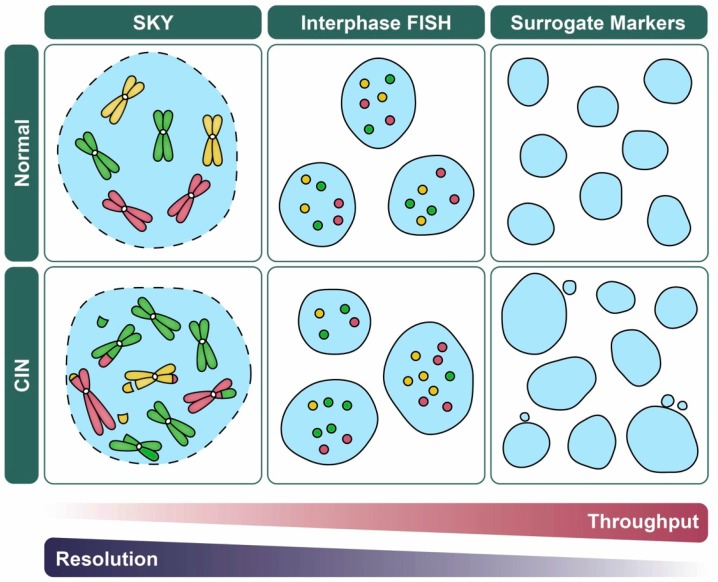
CIN-based analytics and the balance between data throughput and resolution. Schematic illustrating various quantitative imaging approaches used to assess CIN and their strengths in data throughput versus resolution (i.e., N- versus S-CIN). For illustrative purposes, representative examples of normal (top) and CIN-positive (bottom; N- and S-CIN) cellular contexts are shown. FISH (Section 4.2) and related approaches (e.g., fluorescent operator/reporter systems; Section 3.2) rely on enumeration of fluorescent foci rather than whole chromosomes which facilitates manual or automated analyses, but they do not detect N- or S-CIN involving other loci. Spectral karyotyping (SKY; Section 4.3) enables a genome-wide assessment of N- and S-CIN, but is not typically used to assess large sample sizes. Surrogate markers of CIN including changes in nuclear area and micronucleus formation (Section 5.1), are amenable to high-throughput approaches, but they do not specifically assess N- or S-CIN, and they may be subject to false positive/negative results.

FISH is a powerful tool used to evaluate cytogenetic abnormalities that involves the hybridization of a fluorescently labeled probe to a specific gene, region (e.g., centromere enumeration probe; CEP) or to the whole chromosome. Since FISH can be applied to both mitotic and interphase populations, it enables the analysis of cell-to-cell heterogeneity, and it is ideally suited to CIN-based studies (Table 1). In general, both the number and size of FISH signals can be quantified and they can provide insight into the nature of chromosomal aberration [90,91,92]. Under normal conditions, two FISH signals/gene or chromosome are expected in a diploid cell and deviations from this number are suggestive of CIN (Figure 4). For example, increases in the number or sizes of FISH signals are indicative of copy-number gains (e.g., whole chromosome gain (N-CIN) or gene amplification (S-CIN)), while decreases in the number or sizes of FISH signals are indicative of copy number losses (e.g., whole chromosome loss (N-CIN) or gene deletion (S-CIN)) or segmental deletions (S-CIN), respectively. A fundamental benefit of FISH is that several probes can be multiplexed to concurrently assess multiple genes, regions, or chromosomes [2,38,87]. For example, Shiroma and colleagues multiplexed *KRAS* and centromere (chromosome 12) probes to correlate gene amplification (S-CIN) and whole chromosome copy number changes (N-CIN) with tumor stage, grade, and survival in pancreatic ductal adenocarcinomas [93], while Penner-Goeke et al. multiplexed three distinct CEPs to determine the prevalence and dynamics of N-CIN within recurrent and drug-resistant high-grade serous ovarian cancer [94]. Unfortunately, multiplexing is typically limited to ≤ 4 probes due to the limited number of distinct fluorophores that can be individually imaged without spectral overlap on most conventional fluorescent microscopes [94,95]. Finally, while gene-specific and regional FISH-based approaches have been employed to identify N- and S-CIN, they are inherently incapable of identifying N- and S-CIN events not involving the specific probes employed [38,87,96]. In these instances, whole chromosome probes/paints may prove more effective at identifying instances of N- and S-CIN as they label larger portions of the genome, but they are also limited in their ability to only identify events involving the labeled chromosomes. Nonetheless, these approaches have been successfully employed in both research and clinical settings, including both solid and hematological (liquid) cancers [95,97].

### 4.3. Spectral Karyotyping and Multiplex-Banding Techniques

SKY and M-banding are advanced chromosome painting (FISH-based) techniques that afford detailed views of the precise chromosomal alterations occurring within a given cell, and thus can resolve both N- and S-CIN (Table 1) [98,99,100]. Both techniques employ multiple fluorochrome probes that are hybridized to mitotic chromosomes. SKY offers full genome coverage using five distinct fluorochromes in various combinations to paint each chromosome a unique color (Figure 3). This allows for a comprehensive and simultaneous assessment of all chromosomes that readily identifies N-CIN and some S-CIN; provided that the alterations (translocations, amplifications, and deletions) are of sufficient size (typically >1 Mb), intrachromosomal inversions are challenging to identify. On the other hand, M-banding employs multi-colored, region-specific probes to generate unique banding patterns for a specific chromosome or chromosomal region, and thus it is ideally suited to identify S-CIN events, including intrachromosomal inversions (Figure 3) [98,100]. Although these approaches provide insight into N- and S-CIN, they require specialized fluorescent microscopes and software to properly acquire and analyze the multi-channel fluorescent images [98,99,100]. Nevertheless, both SKY and M-banding have been used in numerous cancer contexts, including cell lines and patient samples [98,99,100]. For example, Conde et al. employed SKY to determine the impact that increased Survivin expression has on CIN and tumorigenicity in glioma cell lines [99], while Lettessier et al. used M-banding to characterize recurrent and rare chromosome abnormalities found in breast cancer cell lines [100].

## 5. Quantitative and High-Throughput Imaging Cytometry

In certain contexts, the ability to quantify single cell data from a large number of cells or conditions (e.g., when performing large-scale chemical or genetic screens) may be more relevant than the ability to determine the precise numerical and structural chromosomal changes present within a given sample (Figure 4). In this regard, quantitative imaging cytometry (i.e., measurement of cellular properties) is ideally suited to enhance experimental throughput, particularly when coupled with automated analysis. In addition, sample size is an important consideration, and it becomes critical when assessing the cell-to-cell heterogeneity contained within CIN-positive samples [101]. Here, we discuss two quantitative, high-throughput imaging cytometry approaches used to assess CIN: (1) QuantIM (Section 5.1); and (2) imaging flow cytometry (IFC; Section 5.2).

### 5.1. Quantitative Imaging Microscopy

Rather than quantifying specific changes associated with N- or S-CIN, a more rapid way to assess CIN is to quantify phenotypes or surrogate markers of CIN. In this regard, QuantIM is highly effective at identifying significant changes in nuclear areas [73,94,102,103,104] and micronucleus formation (MNF; small DNA-containing bodies found outside the primary nucleus) (Table 1) [50,70,105,106]. Conceptually, changes in nuclear areas (increases or decreases) are typically associated with large scale changes in chromosome numbers. Consistent with this notion, many studies in various cancer types have identified a positive correlation between nuclear area and DNA content [102,107,108,109]. Accordingly, an increase in the heterogeneity associated with nuclear areas relative to controls is suggestive of CIN. Indeed, changes in nuclear areas have been employed as effective screening tools to uncover CIN in numerous cellular contexts, including human cell lines [73,94,102,103] and primary patient samples [94,104]. For example, significant increases in nuclear areas and CIN were observed in serial samples that are isolated from women with recurrent and drug-resistant ovarian cancer [94]. On the other hand, micronuclei typically arise from chromosome missegregation events involving whole chromosomes or large fragments that fail to incorporate within daughter nuclei following entry into G1 [110]. Micronuclei are observed in a variety of cancers [111,112,113] and they are believed to arise following genotoxic stress; they are also classical hallmarks of CIN [2,106]. Increases in micronucleus formation are associated with CIN in cervical cancer cells following down-regulation of NOP53, a key protein that is involved in tumor suppression and oncogenesis [55].

There are a number of benefits that are associated with nuclear area and micronucleus formation assays: (1) they can be performed in live or fixed cells, (2) they are low-cost, and (3) they are simple to execute, as they require labeling with only a standard DNA counterstain (DAPI or Hoechst). Further, these assays are easily adapted to high-content (multiplexed), high-throughput screens that can be automated with respect to experimental execution and downstream analyses (Figure 4). However, changes in nuclear areas and micronucleus formation can also occur independent of CIN. For example, nuclear areas vary throughout the cell cycle, as G1 nuclei (pre-replication) are smaller than G2 nuclei (post-replication); however, the differences observed between G1 and G2 are typically smaller than those that occur in CIN-positive cells [70]. Additionally, as cells progress through the cell cycle, chromosomes contained within a micronucleus may reintegrate within the primary nucleus as cells progress through a subsequent round of mitosis [111]. Overall, the quantitative assessment of nuclear areas and micronucleus formation are indirect methods of evaluating CIN that are ideally suited to initial screens that will warrant further complementary approaches to validate N- and/or S-CIN phenotypes.

### 5.2. Imaging Flow Cytometry

Unlike most other approaches that demand a compromise between detail/complexity and throughput (Figure 4), IFC is a promising technological advancement that is capable of reconciling these seemingly conflicting properties (Table 1). In essence, IFC combines flow cytometry with high-speed image capture to offer substantial analytical power (thousands of cells/second) combined with the accuracy and resolution of QuantIM techniques [101]. Like traditional flow cytometry, IFC is typically performed by using a fluorescently labeled population of cells in suspension that undergo laser excitation as they pass through a flow cytometer. However, as IFC also combines image capture with downstream image analysis, it enables novel spatial resolution capabilities [114] that can detect CIN-associated phenotypes such as changes in nuclear areas [115] or micronucleus formation [116]. Further, cells can also be labeled with FISH probes and gains/losses in specific signals (e.g., CEPs) can be rapidly quantified as detailed above (Section 4.2) [117,118]. Thus, IFC provides many of the same benefits described for FISH and QuantIM, and in some cases it offers additional advantages over these techniques. In fact, IFC coupled with FISH performed in suspension is proposed to improve the signal-to-noise ratio relative to traditional FISH, which enhances the downstream image analyses [117]. For example, Worrall et al. employed CEP labeling and IFC within human retinal pigment epithelial cells, and identified differential missegregation rates for different chromosomes, with particularly elevated missegregation rates being observed for chromosomes 1, 2, and 3 [117]. As with QuantIM, IFC is amenable to multiplexed analyses involving several phenotypes (nuclear areas or micronucleus formation) or FISH probes simultaneously. In general, IFC is compatible with a variety of sample types, including suspension and adherent cell lines [119], along with hematological cancers [120] or circulating tumor cells [121,122]. The application of IFC in solid tumor samples has not yet been proven, but it is expected to function similarly to standard flow cytometry. Cost is a fundamental limitation of this approach as specialized equipment is required; however, despite this limitation, IFC is an emerging tool with a tremendous potential to evaluate CIN, in both research and clinical settings.

## 6. Single-Cell Genomic Approaches

Recent scientific advances have allowed genomic analyses to be conducted at the single-cell level, enabling the assessment of both N- and S-CIN at an unprecedented resolution. These single-cell genomic approaches include single-cell CGH (sc-CGH), single-cell whole-exome sequencing (sc-WES), single-cell whole-genome sequencing (sc-WGS) and single-cell copy number variation (sc-CNV) analyses. Currently, most single-cell genomic approaches require that single cells be isolated, and their DNA amplified prior to any subsequent analysis; thus, a common limitation of this approach is that not all regions of the genome may amplify in an identical fashion. A variety of single cell isolation and DNA amplification techniques are currently available, and these approaches continue to evolve to improve capture rates of rare cells (e.g., circulating tumor cells) and increase the accuracy of DNA amplification [123,124]. Additionally, novel single-cell whole-genome approaches are beginning to emerge that do not require preamplification of DNA [125,126].

### 6.1. Single-Cell Comparative Genomic Hybridization

Sc-CGH is similar to traditional CGH in that it compares hybridization signal intensities between test (e.g., cancer cells, fluorescently labeled red) and reference (e.g., normal cells, fluorescently labeled green) conditions to identify both gene and whole chromosome CNVs (Table 1) [127]. Briefly, following DNA isolation, test and reference samples are differentially fluorescently labeled (red or green, respectively), denatured and hybridized in a single reaction so that copy number variations can be identified within the test sample relative to the diploid (normal) control. For example, the presence of green signals are indicative of increases in test sample copy numbers, while increases in red signals are indicative of copy number losses within the test sample; yellow signals identify regions of parity. However, unlike traditional CGH, which pools DNA from millions of cells, sc-CGH compares gene/chromosome copy numbers on a cell-by-cell basis [65]. As a result, sc-CNV is capable of identifying cell-to-cell heterogeneity and can distinguish both N- and S-CIN, provided that the events are large enough (typically at least ≥8.3 Mb) [128]. In 2007, Fiegler et al. employed sc-CNV to evaluate CIN in a primary renal adenocarcinoma cell line and a colorectal cancer cell line. They showed that sc-CGH technology effectively identifies cell-to-cell heterogeneity and suggest it may be useful for clinical diagnostics [128].

### 6.2. Single-Cell Whole-Exome Sequencing and Single-Cell Copy Number Variation Analysis

Sc-WES evaluates all protein coding regions within a single cell and is capable of detecting N- and S-CIN (Table 1) [129]. However, as only ~2.0% of the human genome codes for proteins, sc-WES provides limited insight into S-CIN, and may miss critical events having significant implications in disease pathogenesis [130]. Nonetheless, sc-WES can be coupled with downstream CNV, which are commonly annotated using a read depth-based approach (detailed in [129]). Importantly, sc-WES is able to identify intrachromosomal CNVs (S-CIN) provided the events are at least 1 Mb in size, and it can uncover chromosome copy number changes (N-CIN). Additionally, sc-WES is less expensive, enables a greater sequencing coverage, and requires less complex data analysis compared to sc-WGS [129,130]. Despite this, sc-WGS is widely used in research, and it has provided novel insight into tumor heterogeneity. In 2017, Liu et al. employed multi-region sc-WES to evaluate the extent of intratumoral heterogeneity and CNV profiles in primary rectal tumors of two patients that underwent primary tumor resection [131]. Importantly, this study identified remarkable heterogeneity in the tumors from both patients, suggesting intratumoral heterogeneity may be prevalent in rectal cancer [131]. Similarly, Wu et al. used traditional WES and sc-WES on DNA isolated from sporadic colorectal cancer patients, and showed that different regions within each individual tumor contained distinct subpopulations and levels of heterogeneity [132].

### 6.3. Single-Cell Whole-Genome Sequencing and Single-Cell Copy Number Variation Analysis

Sc-WGS evaluates the entire genome within a single cell (Table 1) [131,132] and as with sc-WES can be coupled with sc-CNV. In general, sc-CNV can be annotated in a variety of ways, including: (1) paired-end mapping, (2) split read mapping, (3) read depth, (4) de novo assembly of a genome, and (5) a combination of the above approaches, which are all described in detail elsewhere [130]. Importantly, both paired-end and split read mapping have significant advantages in resolution as they can identify S-CIN less than 1 kb in size. More specifically, paired-end mapping has the ability to identify small structural variants, like insertions, deletions, and duplications that range from 10–100 bp, while the split read mapping can identify breakpoints of large deletions (up to 10 kb) and medium-size insertions (as small as 20 bp) [130]. However, these analytical tools cannot accurately evaluate exact copy numbers and are not applicable to large insertions >1 kb. An advantage of the read depth approach is that it can identify intrachromosomal CNVs (S-CIN) provided the events are >1 kb. It can also identify exact copy number changes (N-CIN) similar to sc-WES, but it is limited in its ability to predict precise breakpoints, or copy neutral events such as translocations and inversions [130]. On the other hand, de novo assembly of a genome allows for the discovery of novel mutation sequences, as it does not employ a reference genome as input, but this requires extensive computation and performs poorly on highly repetitive regions [130]. A fundamental limitation of sc-WGS is the resulting data can be challenging to annotate due to tumor complexity, contamination by normal tissue, and sequencing noise/biases [129]. To overcome these limitations, a combination of approaches is often employed to increase CNV detection. Importantly, sc-WGS paired with CNV analysis allows researchers to identify precise chromosomal alterations at an unmatched resolution. For example, Greene et al. employed sc-WGS and sc-CNV to estimate chromosome instability in circulating tumor cells isolated from metastatic castration-resistant prostate cancer patients [133], while Gao and colleagues employed sc-WGS to evaluate both N- and S-CIN in circulating tumor cells from colorectal cancer patients, and described the evolutionary process of CNVs leading to metastases [134].

## 7. Conclusions

CIN drives the development and progression of the majority of cancers, and it has significant implications for the acquisition of multi-drug resistance [18] and in therapeutic targeting [38,59]. Despite the importance of CIN and its relevance and impact in oncogenesis, there remains a paucity of information as to the genes and pathways that are normally responsible for regulating chromosome stability in humans. Furthermore, detection of CIN within a clinical setting is likely to provide valuable prognostic information and inform treatment decisions, as CIN represents a therapeutic vulnerability that may be common to a large number of cancer patients [38]. Thus, having reliable tools and methods to accurately detect and quantitatively assess CIN is critical to greatly expand our current understanding of the etiological origins driving CIN in both research and clinical settings. Importantly, CIN describes a rate of change in chromosome complements, and it must be evaluated using techniques that are capable of capturing its dynamic nature in live cells, or the cell-to-cell heterogeneity resulting from the ongoing process of CIN. Here we have discussed many conventional and emerging technologies that are used to detect both N- and S-CIN. We have highlighted their strengths and limitations and provided brief examples of how they are used within research and/or clinical contexts. For those interested in assessing CIN or identifying CIN genes, selecting the most appropriate technique to employ will ultimately depend upon the setting (i.e., research or clinical) and the experimental question, particularly as it pertains to N- or S-CIN. In general, we expect that a greater versatility of tools for identifying and evaluating CIN will promote a better understanding of the role CIN plays in cancer that will be critical to ultimately improve the lives and outcomes of those living with cancer.

## Figures and Tables

**Table 1:** Summary of the approaches that are used to evaluate CIN.

Approach	N-CIN ^1^	S-CIN	Principle & Benefits	Limitations
Live-Cell Approaches and Transgenic Chromosome Markers				
Fluorescent labeling of chromatin-associated proteins	**(X)**	**(X)**	Can monitor chromosome dynamics and detect aberrant mitotic events; amenable to automation and multiplexing ^2^.	Requires the generation of a transgenic cell line; specific chromosomes cannot be directly enumerated.
Fluorescent reporter/operator systems	**X**		Can detect specific chromosomes and track gains/losses through cell division; amenable to automation and multiplexing.	Requires the generation of a transgenic cell line; risk of disrupting endogenous sequences.
Human & mouse artificial chromosomes	**X**		Enables the detection of N-CIN without disruption of endogenous sequences; amenable to automation and multiplexing.	Requires the generation of a transgenic cell line; does not directly detect changes involving endogenous chromosomes.
Modified gene editing systems	**X**	**X**	Enables the labeling of precise genomic loci; may be adapted for labeling whole chromosomes.	Requires the generation of a transgenic cell line; techniques are not fully developed.
Cytogenetic Approaches				
Giemsa banding & inverted DAPI ^3^ counterstaining	**X**	**X**	Provides a whole-genome assessment of N-CIN and large-scale S-CIN events.	Small-scale S-CIN events may be missed; requires dividing cells; analysis of chromosome banding patterns can be challenging.
Fluorescence in situ hybridization (FISH)	**X**	**X**	Enables the labeling of specific genes, regions, or whole chromosomes; can be applied to fixed samples and/or dividing cells; amenable to automation and multiplexing.	Only detects events involving the labeled chromosomes; multiplexing depends on the availability of spectrally distinct fluorophores.
Spectral karyotyping & multiplex-banding	**X**	**X**	Provides full genome assessment and/or enhanced resolution of intrachromosomal S-CIN events.	Requires dividing cells; requires specialized equipment; typically used to assess small sample sizes.
Quantitative and High-Throughput Imaging Cytometry				
Quantitative imaging microscopy	**(X)**	**(X)**	Enables the detection of CIN-associated phenotypes using simple DNA counterstaining; low-cost; amenable to automation and multiplexing.	No direct visualization of chromosomes; requires subsequent validation.
Imaging flow cytometry	**X**	**X**	High-throughput method that can be paired with FISH; amenable to automation and multiplexing.	Equipment can be costly and is highly specialized.
Single Cell Genomics				
Sc-CGH, sc-sequencing	**X**	**X**	Provides a high resolution of copy number alterations that are present in individual cells.	May require specialized cell-capture and DNA amplification techniques; data analysis can be complex.

^1^ X, directly detects numerical (N-CIN) or structural (S-CIN) chromosome alterations; (X), detects CIN-associated phenotypes from which N- or S-CIN is inferred. ^2^ Multiplexing is defined as performing multiple concurrent assays/assessments. ^3^ 4′,6-diamidino-2-phenylindole (DAPI).
